# How Physiological Temperature Drives Ligand Recognition and Channel Gating

**DOI:** 10.1063/4.0000990

**Published:** 2025-10-27

**Authors:** Wei Lu

**Affiliations:** Northwestern University, Evanston, IL, USA

## Abstract

Temperature profoundly affects macromolecular function, particularly in proteins with temperature sensitivity. However, its impact is often overlooked in biophysical studies typically performed at non-physiological temperatures, potentially leading to inaccurate mechanistic and pharmacological insights. Using single-particle cryo-electron microscopy at physiological temperature, we investigated temperature-sensitive ion channels TRPM4 and TRPM3, uncovering thermally driven conformations and modes of ligand recognition. In TRPM4, we discovered a “warm” conformation driven by a temperature-dependent Ca²^+^ binding site in the intracellular domain (ICD), essential for physiological gating. Ligands such as decavanadate (a positive modulator) and ATP (an inhibitor) bind to different sites at physiological temperature than at lower temperatures, revealing a previously unrecognized dimension of ligand recognition. Structural snapshots captured at physiological temperature also revealed the elusive open state of TRPM4, not observed at cryogenic preparation conditions. We extended these findings to TRPM3, a key nociceptor for sensing noxious heat. TRPM3 shares a convergent activation mechanism in which both heat and a synthetic agonist induce similar ICD rearrangements, shifting the channel toward an activated state. Collectively, our studies identify the ICD as a crictial module for thermal sensing and establish a structural framework for temperature-dependent ligand recognition and modulation in TRPM channels. These findings underscore the importance of studying thermosensitive proteins under physiological temperature to obtain accurate mechanistic and pharmacological insights.

